# The interplay between lipids and dopamine on α-synuclein oligomerization and membrane binding

**DOI:** 10.1042/BSR20130092

**Published:** 2013-10-22

**Authors:** Chi L. L. Pham, Roberto Cappai

**Affiliations:** *Department of Pathology, The University of Melbourne, Victoria 3010, Australia; †Bio21 Molecular Science and Biotechnology Institute, The University of Melbourne, Victoria 3010, Australia

**Keywords:** α-synuclein, dopamine, lipid, oligomer, Parkinson’s disease, CMC, critical micelle concentration, DA, dopamine, DHPC, 1,2-dihexanoyl-*sn*-glycero-3-phosphocholine, DHPS, 1,2-dihexanoyl-*sn*-glycero-3-phospho-L-serine, LUV, large unilamellar vesicle, Met-ox, methionine-oxidized, PD, Parkinson’s disease, POPC, 1-palmitoyl-2-oleoyl-*sn*-glycero-3-phosphocholine, POPS, 1-palmitoyl-2-oleoyl-*sn*-glycero-3-phospho-L-serine, PUFA, polyunsaturated fatty acid, ROS, reactive oxygen species, SUV, small unilamellar vesicle, α-syn, α-synuclein

## Abstract

The deposition of α-syn (α-synuclein) as amyloid fibrils and the selective loss of DA (dopamine) containing neurons in the *substantia nigra* are two key features of PD (Parkinson's disease). α-syn is a natively unfolded protein and adopts an α-helical conformation upon binding to lipid membrane. Oligomeric species of α-syn have been proposed to be the pathogenic species associated with PD because they can bind lipid membranes and disrupt membrane integrity. DA is readily oxidized to generate reactive intermediates and ROS (reactive oxygen species) and in the presence of DA, α-syn form of SDS-resistant soluble oligomers. It is postulated that the formation of the α-syn:DA oligomers involves the cross-linking of DA-melanin with α-syn, via covalent linkage, hydrogen and hydrophobic interactions. We investigate the effect of lipids on DA-induced α-syn oligomerization and studied the ability of α-syn:DA oligomers to interact with lipids vesicles. Our results show that the interaction of α-syn with lipids inhibits the formation of DA-induced α-syn oligomers. Moreover, the α-syn:DA oligomer cannot interact with lipid vesicles or cause membrane permeability. Thus, the formation of α-syn:DA oligomers may alter the actions of α-syn which require membrane association, leading to disruption of its normal cellular function.

## INTRODUCTION

PD (Parkinson's disease) is characterized by the selective loss of dopaminergic neurons in the *substantia nigra* and the formation of intracellular Lewy bodies. While insoluble α-syn (α-synuclein) amyloid fibrils are the most prominent component of the Lewy bodies, various α-syn oligomeric species have been observed including spherical, chain-like, ring-like and annular structures [1,2]. The annular oligomers have pore forming activity by interacting with lipid membrane and causing membrane instability and permeability and in turn causing toxicity [3–8].

α-Syn is a presynaptic, natively unfolded protein of 140 amino acids (14.6 kDa) with an amphipathic N-terminal consist of seven 11-amino acid imperfect repeats with the consensus sequence KTKEGV, a central hydrophobic domain important for fibril formation and the acidic CTD (C-terminal domain). Upon binding lipids, the N-terminal of α-syn undergoes a conformational change into an α-helical structure while the C-terminus remains unfolded [9–12]. Using lipid membrane mimetic models such as SDS micelle and lipid vesicles of different sizes and lipid composition, α-syn displays higher affinity to negatively charged lipids and high curvature SUVs (small unilamellar vesicles) [7,8,13–15]. However, α-syn also binds to LUVs (large unilamellar vesicles) with either anionic or Zwitterionic headgroups [16]. Membrane association is thought to be an important aspect of its function since α-syn is associated with cellular membranes [6,17,18] with approximately 15% bound to membranes in rat brain homogenates [19] and 20–25% associated with membranes in cell culture models [18]. α-Syn interacts with lipid rafts [20], lipid droplets [18] and with mitochondria membranes [21,22]. The interaction of α-syn to mitochondria can induce mitochondrial membrane depolarization and loss of phosphorylation capacity [23]. The binding of α-syn to synaptic vesicles may regulate cytoplasmic DA (dopamine) levels by controlling DA vesicular uptake for storage and/or DA vesicular release into the distal pool of presynaptic terminals [24–26].

Binding to lipid vesicles can modulate the aggregation of α-syn and the effect of lipids on fibril formation is dependent on α-syn conformation. At a low protein to lipid ratio (1:5), α-syn adopts 60–70% α-helical structure and fibril formation is inhibited, while at a high protein to lipid ratio (5:1) α-syn adopts a partially folded conformation with accelerated fibril formation [27]. The interaction between α-syn and PUFA (polyunsaturated fatty acid) induces oligomerization [28,29] and fibril formation [29,30]. Oligomerization of α-syn can occur once bound to lipid membranes [18,19] and α-syn aggregate on the lipid bilayer [31,32]. At a 1:50 ratio of protein to lipid, α-syn undergoes conformational change from α-helical monomeric conformation to β-sheet fibrils [32] and in doing so, extract lipids from membrane and disrupt membrane integrity [31]. High affinity lipid-binding correlated with an intermediate formed during the early phase of α-syn aggregation [15].

DA and its oxidative intermediates can induce the formation of SDS-resistant soluble α-syn oligomers [33–37] that are off-pathway to fibril formation [36]. DA causes the oxidation of all four methionine residues in α-syn [36,38]. The solution and structural properties of these oligomers indicated that the α-syn:DA oligomer are composed of overlapping worm-like monomers with little structural content, and held together by the DA-melanin polymer, most likely via interactions with the lysine and tyrosine residues [39]. Given the importance of lipids upon the properties of α-syn, this study investigated the role of lipids in relation to the DA-mediated α-syn oligomerization. Firstly, we determined the effect of lipids on the ability of DA to modulate α-syn oligomerization and secondly, we studied the lipid binding properties of the α-syn:DA oligomer.

## MATERIALS AND METHODS

### Materials

Lipids were purchased from Avanti Polar Lipids Inc. DA hydrochloride was purchased from Sigma-Aldrich. SDS/PAGE analysis was performed using 12% Bis-Tris gel from Invitrogen. SDS 20% (w/v) solution and the silver stain reagents were from Bio-Rad. All other reagents, solvents, buffer and media components were of analytical grade or of the highest purity available.

### Expression and purification of α-syn

The expression and purification of α-syn from *Escherichia coli* was performed as previously described [34]. Protein purity and identity was accessed by MS and SDS/PAGE analysis. The protein concentration was determined using the extinction coefficient of 5120 M^−1^ cm^−1^ at absorbance 280 nm.

### Formation of DA-induced α-syn oligomers in the presence of lipids

Lyophilized α-syn was prepared for oligomerization experiment as previously described [36]. Sample of freshly prepared α-syn (20 μM) were incubated at 37°C for 16 h with and without DA in the presence of various concentration of SDS, short chain phospholipids (DHPC (1,2-dihexanoyl-*sn*-glycero-3-phosphocholine) and DHPS (1,2-dihexanoyl-*sn*-glycero-3-phospho-L-serine)) and liposome as stated in figure legends. Soluble oligomers were obtained by ultracentrifugation in a Beckman TL-100 ultracentrifuge at 390880 ***g*** (100000 rpm) for 20 min at 4°C. Supernatants were collected for SDS/PAGE/silver staining analysis and for CD spectroscopy measurements.

### Preparation of α-syn oligomers formed in the presence of DA

α-Syn Met-ox (methionine-oxidized) monomer and α-syn:DA oligomers were formed and purified as described [39]. To obtained high concentrations for characterization, the proteins were concentrated using a spin column with MWCO 3,000 Da (Millipore). BCA protein assay was performed to determine protein concentration of Met-ox monomer and α-syn:DA oligomers, using un-oxidized α-syn as a standard protein.

### Preparation of liposomes

Liposomes were composed of POPC (1-palmitoyl-2-oleoyl-*sn*-glycero-3-phosphocholine), or POPS (1-palmitoyl-2-oleoyl-*sn*-glycero-3-phospho-L-serine) or a 1:1 mixture of POPC and POPS were prepared. Lipids were dissolved in chloroform and dried under nitrogen gas, forming a film. This film was rehydrated in 10 mM sodium phosphate pH 7.4 to a concentration of 10 mM and incubated at 37°C with shaking at 200 rev./min for 2 h, in the presence of glass beads. The lipid suspension was subjected to 5 freeze-thaw cycles. The lipid solution was extruded at least 17 times using a ‘mini-extruder’ apparatus (Avanti). For preparation of LUV, lipid solution was passed through a 100 nm pore filter (Millipore). For making SUV, after extrusion through 100 nm pore filter, the sample was then extruded through a 50 nm pore filter. Lipid vesicles were purged with N_2_ gas and stored at room temperature and used with 24 h of preparation.

### Preparation of calcein encapsulated liposomes and permeabilization assay

Calcein encapsulated liposome was prepared as described above for the preparation of LUV particle. However, a solution of 70 mM calcein in PBS buffer was used to rehydrate the lipid film. After the lipid solution was extruded, free calcein was removed by gel filtration using a column (1 cm ID, 15 cm in length) packed with Sephadex G-200 (superfine) resin (GE Healthcare). Fraction containing LUV particle was diluted 20-fold for use in permeability assay.

Calcein encapsulated within the LUV has a high local concentration causing it to self-quench and thus, exhibit low fluorescence intensity with excitation at 490 nm. Upon release from the liposome environment there is an increase in fluorescence intensity. Disruption of LUV by treatment with 1% Triton, causes total calcein release and a maximum increase in fluorescent intensity. Samples of α-syn were incubated with calcein-encapsulated LUVs for 10 min at room temperature and the emission spectra collected with excitation at 490 nm. The degree of permeability is a measure of the percentage of dye release upon treatment with protein compared with total release of calcein upon treatment with 1% Triton (deemed 100%).

### CD spectroscopy

CD spectra were obtained using a Jasco 810 spectropolarimeter at 20°C. Far-UV CD spectra were obtained from 185 to 260 nm with a 0.1 cm path length quartz cuvette. Spectra were corrected for any contribution due to scattering of lipids. Raw CD signal were normalized to protein concentration and expressed as mean residue ellipticity.

## RESULTS

### The effect of SDS on DA mediated α-syn oligomerization

**Figure 1:**
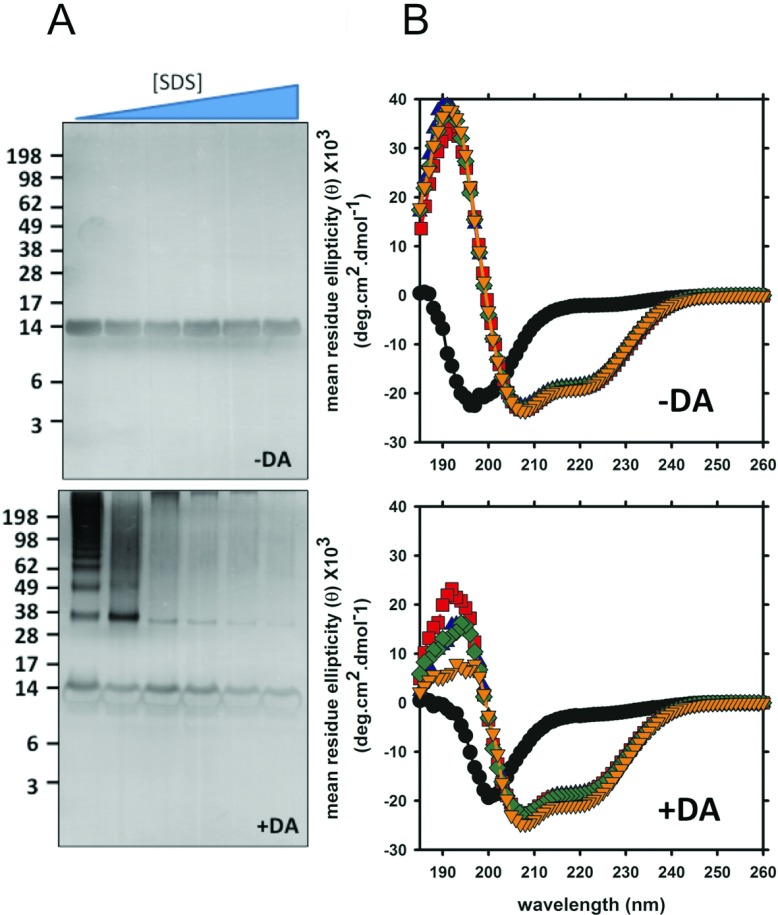
SDS inhibits α-syn oligomerization in the presence of DA α-Syn was incubated at 37°C for 16 h with and without DA in the presence of various concentration of SDS. Soluble fractions were collect after high speed centrifugation and SDS/PAGE (**A**) and CD analysis (**B**) were performed. Concentration of SDS used in sample incubations are 0 mM (lane 1, circles), 1.45 mM (lane 2), 2.89 mM (lane 3, triangles up), 8.67 mM (lane 4, squares), 17.34 mM (lane 6, diamonds) and 34.68 mM (lane 7, triangles down).

SDS is an anionic amphiphilic detergent that can act as a lipid mimetic for studying protein-lipids and protein-membrane interactions. α-Syn in the presence of DA formed SDS-resistant soluble oligomers [34] that accompany the oxidation of four methionine residues [36,38]. Structurally, α-syn adopts a ‘broken helix’ conformation when it interacts with SDS micelles [40,41]. The presence of SDS at low concentrations (0.5–0.75 mM) can accelerate fibril formation while above 2 mM SDS, fibril formation is inhibited [42]. We initially investigated the effect of SDS on DA mediated α-syn oligomerization. The CMC (critical micelle concentration) of SDS in water is 8 mM. α-Syn (20 μM), with and without DA, was incubated in the presence of SDS at various concentrations, ranging from sub-micellar to micelle (1.45–34.68 mM) for 16 h at 37°C. SDS/PAGE/silver staining analysis showed that SDS inhibits DA mediated α-syn oligomerization (Figure 1) in a dose-dependent manner. At sub-micellar SDS concentration (1.45 mM) in the presence of DA, monomeric and dimeric species were observed along with a small amount of higher MW α-syn:DA oligomers. Higher concentrations of SDS (2.89 mM to 34.68 mM) caused total inhibition of α-syn:DA oligomerization (Figure 1A). MS analysis showed oxidation of methionine residues in the α-syn monomeric species incubated with DA, with or without SDS, indicating that SDS does not prevent DA mediated oxidation of α-syn. CD analysis showed α-syn still adopted a classical α-helical conformation in the presence of SDS with DA (Figure 1B). Therefore, DA-mediated oxidation of α-syn did not change the biophysical properties of α-syn.

### The effect of short chain phospholipids on the modulation of α-syn oligomerization by DA

**Figure 2:**
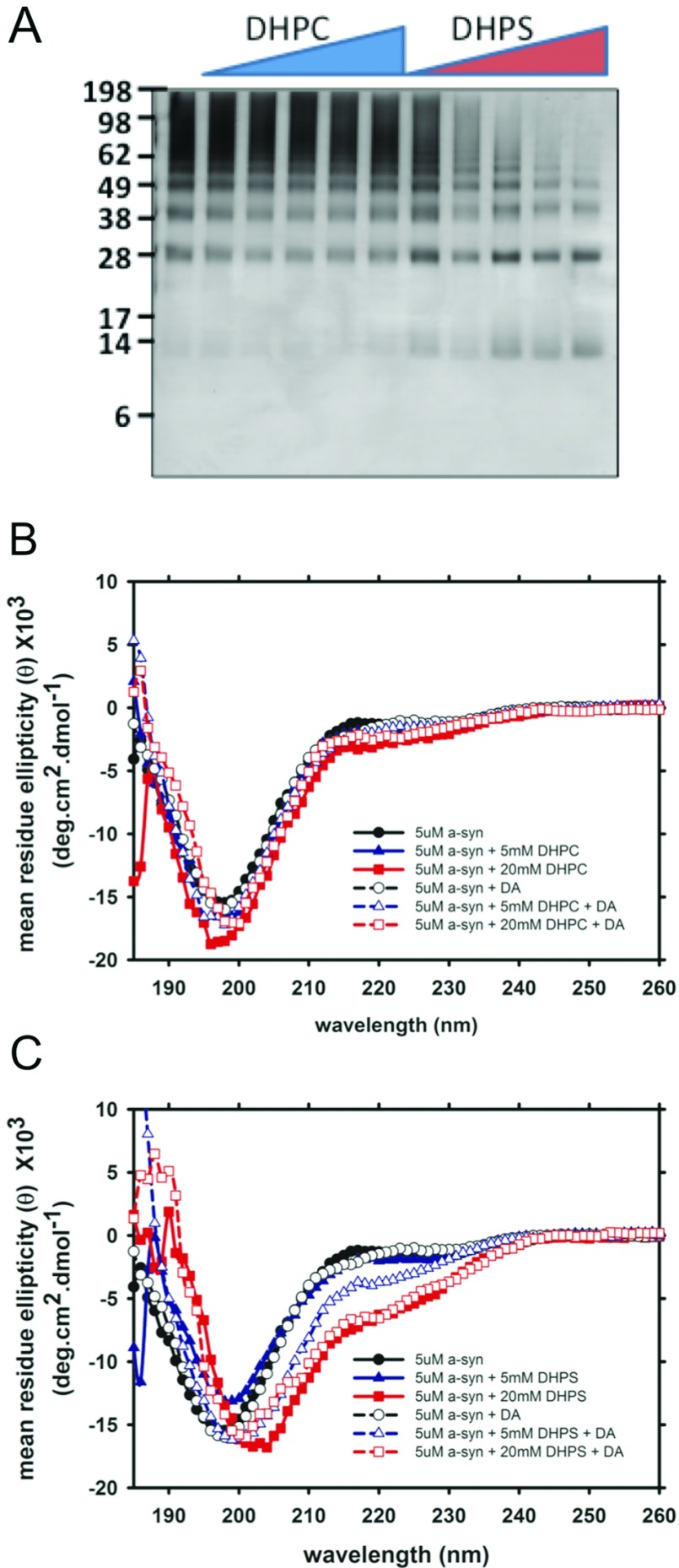
The effect of short chain phospholipids on α-syn oligomerization in the presence of DA (**A**) SDS/PAGE analysis of α-syn incubated at 37°C for 16 h with and without DA in the presence of DHPC or DHPS at various concentration: 0 mM, 2.5 mM, 5 mM, 10 mM, 15 mM and 20 mM. CD analysis of sample of α-syn incubated with (dashed line, opened symbols) and without (solid line, closed symbols) DA in the presence of 0 mM (circles), 5 mM (triangles up) and 20 mM (squares) DHPC (**B**) or DHPS (**C**).

We tested if short chain phospholipids affected DA mediated α-syn oligomerization. Zwitterion charged DHPC (CMC 10 mM) had no effect on DA mediated α-syn oligomerization (Figure 2A); however, DHPS (CMC 15 mM), a negatively charged short chain phospholipid, caused a decrease (from sub-micellar to micellar) in α-syn oligomerization in the presence of DA (Figure 2A). CD analysis showed no conformation change for α-syn in the presence of DHPC, with or without DA. However, there was a noticeable change in the spectra for α-syn in the presence of 20 mM, micellar, DHPS in the absence of DA. The addition of DA caused a slight conformation change in α-syn with sub-micellar 5 mM DHPS. Increasing the lipid ratio to 20 mM micellar DPHS caused further increases in secondary structure in the presence of DA (Figure 2B). The conformational change observed for α-syn in the presence of DHPS is similar to that observed for α-syn incubated with vesicles at 5:1 protein to lipid ratio [4], and was attributed to a partially folded intermediate that can promote aggregation.

### The effect of liposomes on the modulation of DA mediated α-syn oligomerization

**Figure 3:**
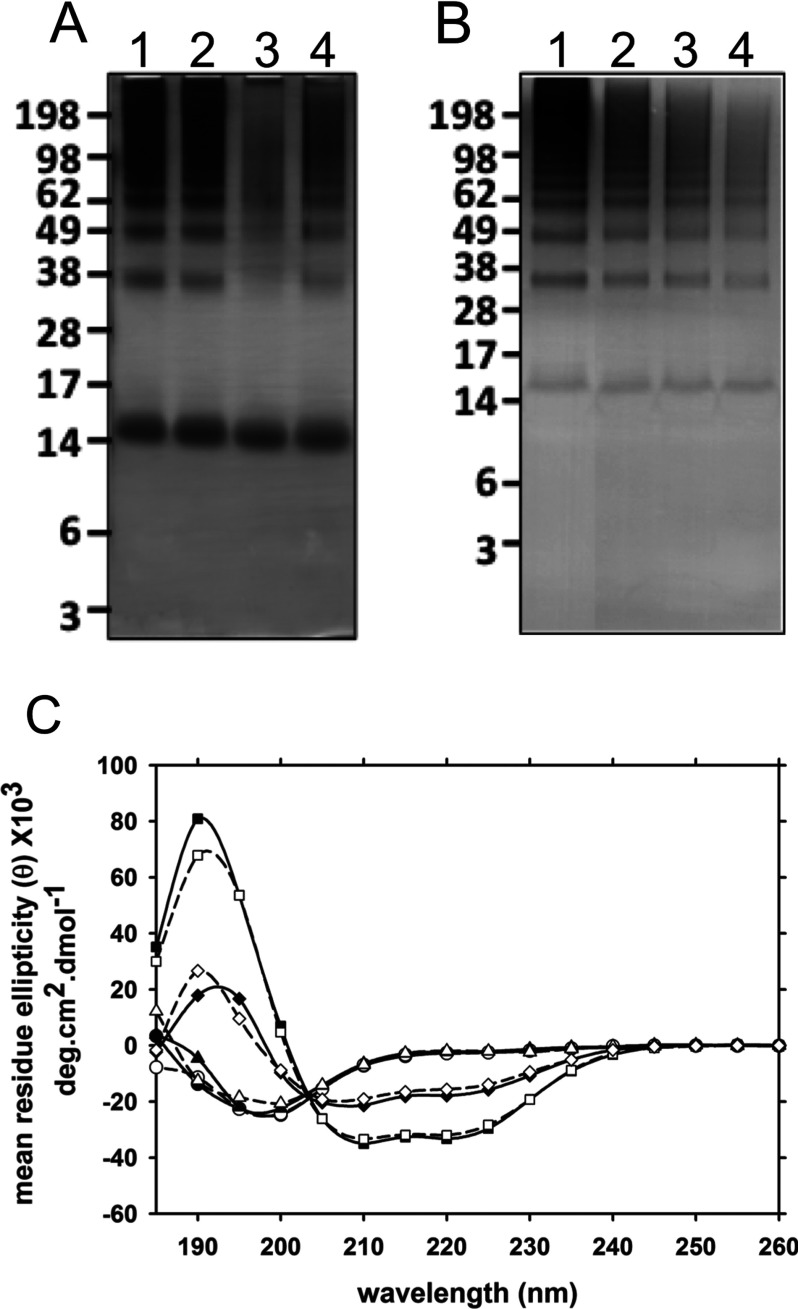
The effect of liposomes on α-syn oligomerization in the presence of DA (**A**) SDS/PAGE analysis of α-syn with DA alone (lane 1) and in the presence of 1 mM POPC LUV (lane 2), POPS LUV (lane 3) and POPC:POPS LUV (lane 4). (**B**) Samples of α-syn with DA alone (lane 1) and in presence of 1 mM (lane 2), 2 mM (lane 3) and 4 mM (lane 4) of POPC:POPS LUV. (**C**) CD spectra of α-syn in the absence (solid line, closed symbols) and presence (dashed line, opened symbols) of DA with No LUV liposome (circles) and with POPC (triangles up), POPS (squares) and POPC:POPS (diamonds).

α-Syn can interact with cellular membranes with preferential binding to negatively charged lipids. We prepared lipid vesicles using Zwitterion charged (POPC) and negatively charged (POPS) phospholipids to study their ability to modulate α-syn oligomerization in the presence of DA. We tested LUVs as a membrane model of organelle such as mitochondria as well as SUVs as a membrane model for synaptic vesicles. Similar to the observation for short chain Zwitterion charge phospholipids, POPC LUV had no effect on the ability of DA to modulate α-syn oligomerization. However, when α-syn was incubated in the presence of POPS LUV and DA, there was a clear decrease in oligomer formation (Figure 3A). Inhibition of α-syn oligomerization was less significant when α-syn and DA were incubated with POPC:POPS LUV. Increasing the vesicle to protein ratios, decreased DA-mediated oligomerization of α-syn (Figure 3B). However, we could not totally inhibit DA-mediated oligomerization by POPC:POPS vesicles. There were no significant differences observed between LUV and SUV in inhibiting DA-mediated oligomerization (results not shown). CD analysis showed no conformation change in α-syn in the presence of POPC LUV, with α-syn remaining in an unfolded conformation (Figure 3C). POPS LUVs (±DA) caused α-syn to adopt an α-helical structure (Figure 3C). An α-helical conformation was also observed for α-syn in the presence of POPC:POPS LUV; however, the α-helical content was less compared with α-syn in the presence of POPS LUV (Figure 3C).

### To determine the lipid binding properties of the α-syn:DA oligomers

**Figure 4:**
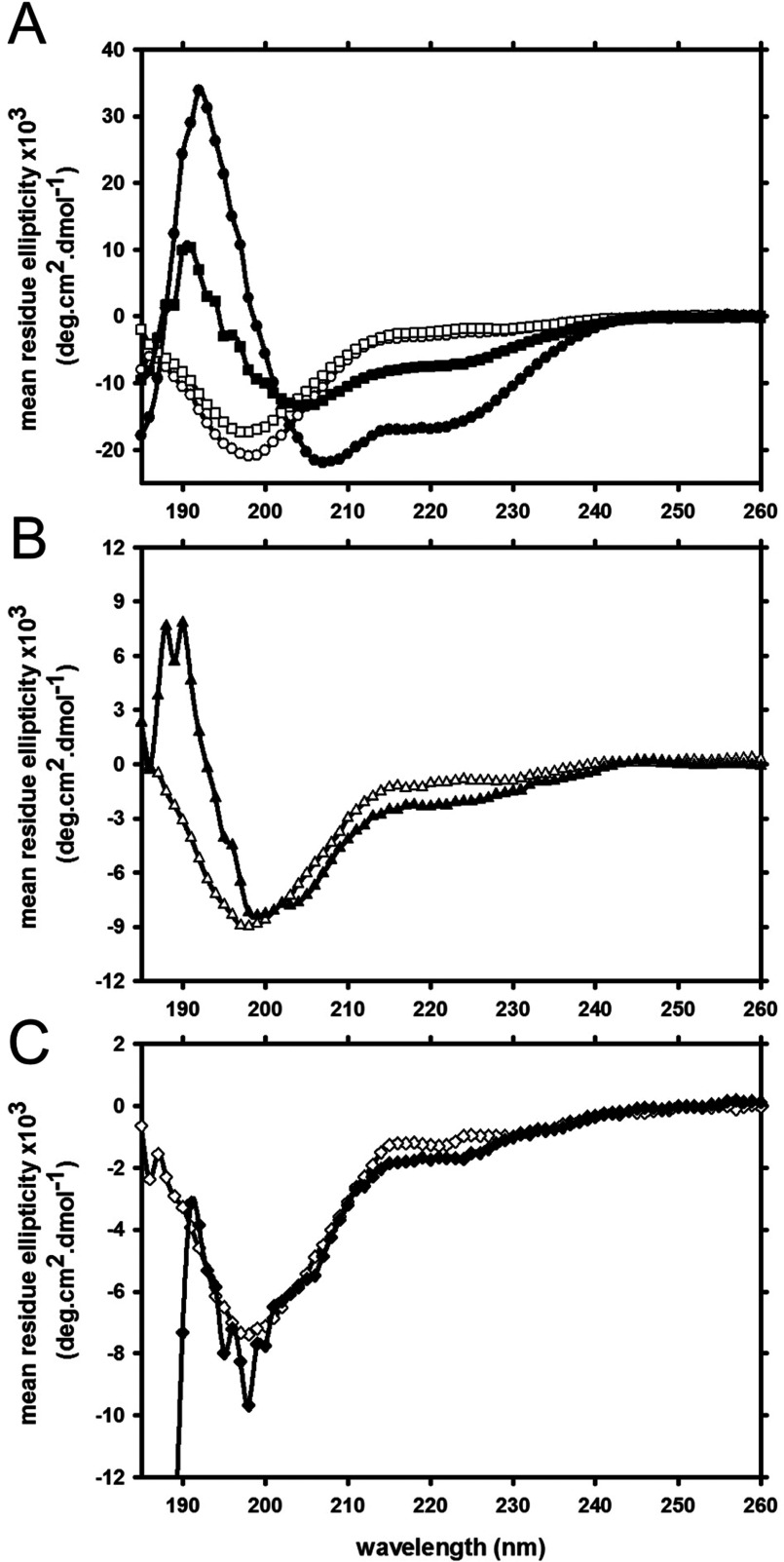
Secondary conformation of α-syn monomer an α-syn:DA oligomers in the presence of lipid vesicles (**A**) CD spectra of un-oxidized (circles) and Met-ox (squares) monomer in the absence (open symbols) and presence (closed symbols) of POPC:POPS LUV. CD Spectra of α-syn:DA dimer (**B**) and α-syn:DA trimer (**C**) in the absence (open symbols) and presence (open symbols) of POPC:POPS LUV.

To determine how the different DA-induced α-syn oligomers interact with lipids, we α-syn incubated with DA and then purified by size exclusion chromatography the Met-ox α-syn monomer, α-syn:DA dimer, trimer species and the higher MW species. Similar to un-oxidized monomeric α-syn, the Met-ox monomeric α-syn was largely unfolded in the absence of lipids and underwent a conformational change to α-helix in the presence of POPC:POPS LUV (Figure 4A). However, there was decrease in helix for the Met-ox α-syn monomer, perhaps reflecting the increase in polarity upon methionine oxidation to form methionine sulfoxides. In contrast, the α-syn:DA dimer (Figure 4B), trimer (Figure 4C) and other higher MW species (results not shown), did not undergo a conformational change in the presence of lipids vesicles and remained unstructured. Therefore, the modification by DA results in species which have distinct biophysical properties to the non-DA generated oligomers, which retain their ability to undergo a conformation change in the presence of lipid [15].

### Membrane permeabilization by DA:α-syn oligomers

Membrane permeabilization may represent a toxic mechanism by α-syn. We tested the membrane permeabilization activity of the α-syn:DA oligomers using a calcein dye-release assay. We isolated, via size exclusion chromatography, the type A1 and A2 oligomers which bind to the A11 oligomer-specific antibody and induce significant membrane permeability [2]. We also purified type A1 and A2 oligomers generated from Met-ox α-syn monomer. The incubation of 5 μM (concentration based on monomer) of un-oxidized α-syn type A1 or A2 oligomer with calcein encapsulate LUV resulted in an increase in fluorescence intensity of 34.8% and 44.9%, respectively, representing calcein dye release into the solvent environment (Table 1). The type A1 and A2 oligomers made from Met-ox α-syn monomer also had the ability to cause membrane permeabilization with a 50.1% and 38.2% dye release for type A1 and A2 oligomers, respectively. Monomeric and fibrillar α-syn (5 μM) induced the release of calcein dye from LUV, but at 15.8% and 8.4%, respectively, significantly lower than the type A1 and A2 oligomers. 5 μM Met-ox monomer caused 18.8% dye release. Increasing the concentration of α-syn monomer increases the percentage of calcein release from LUV (Table 1), with 25.4% dye release for 20 μM α-syn and 33.7% for 20 μM Met-ox monomer. The α-syn:DA dimer and trimer and the higher MW oligomers (20 uM, Table 1) caused less than 15% dye release, indicating that the α-syn:DA oligomers could not effectively induce membrane permeabilization.

**Table 1:** Membrane permeability of α-syn monomer and oligomers Membrane permeability was measured by the release of calcein dye from POPC:POPS LUV (see Materials and Methods). The table shows the% of dye release upon treatment with α-syn monomer and oligomers at various protein concentrations.

	Concentration of protein*		
	5.0 μM	10.0 μM	20.0 μM
α-syn monomer	15.8	21.4	25.4
α-syn fibril	8.4	–	–
α-syn A1 oligomer	34.8	–	–
α-syn A2 oligomer	44.9	–	–
Met-ox α-syn monomer	18.8	24.4	33.7
Met-ox α-syn A1 oligomer	50.1	–	–
Met-ox α-syn A2 oligomer	38.2	–	–
α-syn:DA dimer	12.9	12.9	11.2
α-syn:DA trimer	10.7	11.1	11.9
α-syn:DA HMWO-1	11.7	12.4	13.2
α-syn:DA HMWO-2	11.6	11.9	11.1
α-syn:DA HMWO-3	–	11.4	7.3

*protein concentration is base on monomeric concentration.

HMWO-1, contain a mixture of trimer and tetramer

HMWO-2, contain a mixture of tetramer and pentamer

HMWO-3, contain a mixture of pentamer and hexamer

## DISCUSSION

The formation of small, soluble, oligomeric species have been proposed as a common mechanism in the pathogenesis of protein mis-folding diseases [3]. PD is associated with the loss of dopaminergic neurons and there is a direct neurochemical link between DA and α-syn, with DA mediating the formation of SDS-resistant α-syn soluble oligomers [33–35,43]. The structure, function and cytotoxicity of these oligomeric species is not clearly understood. DA can induce the formation of α-syn oligomers in cultured cells [44–46] and they can be either toxic [45] or non-toxic [44,46]. Oxidative stress, via an increase in ROS (reactive oxygen species), is a potential risk factor for PD and can cause lipid peroxidation resulting in reactive aldehydes and PUFAs that promote α-syn oligomerization [29,47]. Lipid binding is a property of α-syn [9–15], and upon interaction with lipids, it is the N-terminal that is important for the cooperative formation of helical domain in α-syn [9]. Lipids can modulate α-syn oligomerization and fibril formation [16,18,19,27–32]. α-Syn, in the absence or presence of DA, prefers to interact with negatively charged lipids and undergo a conformational change [7,8,13,14].

Our data showed that the negatively charged short chain phospholipid DHPS inhibited the ability of DA to modulate the formation of soluble α-syn:DA oligomers (Figure 2). The interaction between α-syn with lipid vesicles containing the negatively charged phospholipid POPS reduced the formation of α-syn oligomers in the presence of DA (Figure 3) and supports previous studies where co-incubation of α-syn with DA on a lipid bilayer inhibited aggregation [31]. The formation of SDS-resistant oligomers is postulated to occur via cross-linking between DA-melanin and the lysine residues of the imperfect repeats with the consensus sequence KTKEGV at the N-terminal of α-syn [39]. The interaction with lipids occurs at the N-terminal region of α-syn [9] and such lipid-protein interaction may prevent DA-melanin from cross-linking with α-syn to form oligomeric species.

α-Syn oligomers, formed in the absence of DA, are thought to be toxic because they can interact with the lipid bilayer and increase membrane permeability [5]. α-Syn monomers can aggregate into oligomers then fibrils on lipid bilayers, and during the aggregation process it can disrupt membrane integrity [31,32]. Time-dependent SAXS (small-angle X-ray scattering) analysis identified α-syn oligomers to have a wreath shaped structure on pathway to fibril formation, and they are able to permeabilize membranes [48]. Modelling studies demonstrated that monomeric α-syn can rapidly penetrate the lipid bilayer and incorporate additional monomers to form pore-like structures that fully perforate the lipid bilayer [49]. Our present study demonstrated that the presence of lipids vesicle does not induced a conformational change in the α-syn:DA oligomers, suggesting that these oligomers may not interact with lipids or interact in a different manner that does not induce a conformational change. Furthermore, the oligomers did not permeabilize the lipid membrane. It is feasible that the cross-linking of DA-melanin to the N-terminus of α-syn, to form worm-like structures on α-syn:DA oligomers [39], eliminates the availability of the lipid binding sites on α-syn. The DA-melanin cross-linking to the α-syn may be inducing structural inflexibility into the α-syn:DA oligomer, thus preventing it from interacting with lipids to form pore-like structures. These findings have implications regarding the functions of α-syn which require membrane binding. Alterations in cytosolic DA could change the levels of oxidized α-syn, which in turn would alter the membrane binding pool of α-syn thus affecting its actions on synaptic vesicles, mitochondria and the plasma membrane.
